# Decompressive craniotomy in split-technique (DCST) for TBI in infants: introducing a new surgical technique to prevent long-term complications

**DOI:** 10.1007/s00381-024-06445-1

**Published:** 2024-05-24

**Authors:** Sevgi Sarikaya-Seiwert, Ehab Shabo, Arndt-Hendrik Schievelkamp, Mark Born, Christian Wispel, Hannes Haberl

**Affiliations:** 1 Section of Pediatric Neurosurgery, Department of Neurosurgery, Medical Faculty, Rheinische Friedrich Wilhelms University, Venusberg Campus 1, Bonn, D-53127 Bonn, Germany; 2 Department of Neuroradiology, University Hospital Bonn, 53127 Bonn, Germany; 3 Section of Pediatric Radiology, Department of Diagnostic and Interventional Radiology, University Hospital Bonn, Bonn, Germany; 4 Department of Neurosurgery, Schoen Clinic Vogtareuth, Munich, Germany; 5 Department of Neuroradiology, University Hospital Koeln, Koeln, Germany

**Keywords:** Split-technique, Infant, Acute subdural hematoma, Decompressive craniectomy, Hinge craniotomy

## Abstract

**Introduction:**

Decompressive craniectomy (DC) is rarely required in infants. These youngest patients are vulnerable to blood loss, and cranial reconstruction can be challenging due to skull growth and bone flap resorption. On the other hand, infants have thin and flexible bone and osteogenic potential.

**Material and methods:**

We propose a new technique called DCST, which makes use of these unique aspects by achieving decompression using the circumstance of the thin and flexible bone. We describe the surgical technique and the follow-up course over a period of 13 months.

**Results and conclusion:**

In our study, DCST achieved adequate decompression and no  further repeated surgeries in accordance with decompressive craniectomy were needed afterwards.

## Introduction

Different etiological factors can lead to an acute subdural hematoma or raise intracranial pressure in full-term infants. These factors can be congenital malformations, head injury, infection, impaired coagulation, or venous sinus thrombosis. In the adult population with traumatic brain injury (TBI) or ischemic stroke resulting in elevated intracranial pressure, decompressive craniectomy (DC) is an effective surgical method to improve morbidity and prevent mortality. Randomized controlled trials have proven the effectiveness of this procedure [1–3].

Indications for operative management of TBI or raised intracranial pressure in full-term infants are less clear, and surgery is less likely. There is also no consensus about the surgical technique to lower the intracranial pressure to prevent morbidity and mortality [4]. Several surgical techniques are described in the literature including the implantation of an Ommaya reservoir, percutaneous subdural tapping, decompressive craniectomy, hinge craniotomy, endoscopic hematoma aspiration, or barrel stave osteotomy decompression [5, 6]. In the pediatric population, despite a lower degree of evidence, similar feasibility and efficacy of DC are assumed. The infant skull is characterized by open cranial sutures and fontanels. The special skull anatomy allows slow-growing intracranial volumes (e.g., hydrocephalus, benign tumors) to adapt to emerging macrocephaly, but an acute volume increase (such as intracranial hematoma) cannot be compensated by these mechanisms [7]. Large intracranial hematomas in the infant age group are rare, but if they occur, a decompressive craniectomy is necessary and lifesaving [4]. On the other side, cranioplasty after decompressive craniectomy in the pediatric population carries the weight of unsolved problems, mainly due to the visible lack of alternative materials replacing the autologous bone, whose high rate of resorption may sometimes turn it into a liability. Several additional surgical procedures are the consequence [8–12].

We report the management and outcomes of 12 cases of infants with TBI treated successfully by decompressive craniotomy in split-technique (DCST) to prevent long-term complications related to the bone defect after decompressive craniotomy. The introduced technique uses the flexibility of the infant skull and achieves sufficient decompression. In addition, it prevents in our study the need for additional surgeries.

## Material and methods

Twelve cases of infantile TBI and raised intracranial pressure were treated with DCST by the authors. The mean age of the patients was 4.5 months. The main reason for elevated intracranial pressure was an aSDH followed by diffuse brain edema. The article describes the unique surgical technique and the clinical outcome over a period of at least 20 months.

## Results

### Patient collective

All treated patients were infants with a median age of 4.5 months (Table 1). The patients had different disorders leading to an elevation of the intracranial pressure. The main reason was an acute subdural hematoma, followed by a combination of aSDH and intracerebral hemorrhage as well as aSDH in combination with brain edema due to cardiopulmonary resuscitation. All patients showed a midline shift to one side on the trans-fontanel ultrasound of the brain. The doppler-sonography in the anterior cerebral artery showed reduced flow velocities and an increase in the Pulsatility Index (PI) by most of the patients. The following computer tomography (CT) or magnet resonance image (MRI) cross-sections confirmed the midline shift and signs of elevated intracranial pressure.

**Table 1:** Summary of reported cases

**Patient No., sex**	**Age (d)**	**Type of hematoma**	**GCS (before surgery)**	**CT (before surgery)**	**MRI** after surgery	**Treatment**	**Concomitant diseases**	**Follow-up**	**Cause of injury**
1, m	2	aSDH		Yes	Yes	DCST, ICP monitoring	None		Birth trauma
2, m	339	aSDH/iCH, IVH		Yes	Yes	DCST, Rickham-Resevoir	Peritoneal dialysis/nephropathy	Need of VA shunt	Spontaneous
3, f	1	aSDH		Yes	Yes	DCST, ICP monitoring	None		Birth trauma
4, m	327	cSDH+aSDH/brain edema		Yes	Yes	DCST, ICP monitoring	None		Trauma
5, m	2	aSDH/iCH cerebellum		Yes	Yes	DCST, ICP monitoring	None		Trauma
6, f	163	aSDH/cSDH		Yes	Yes	DCST, ICP monitoring	None		Non-accidental injury
7, f	41	aSDH		Yes	Yes	DCST, ICP monitoring	Duodenal, vitamin k malabsorption		Minor trauma
8, f	2	aSDH		Yes	Yes	DCST, ICP monitoring	None		Birth trauma
9, m	332	aSDH/tSAH, brain edema		Yes	Yes	DCST, ICP monitoring	None		Trauma
10, m	355	EDH, aSDH		Yes	Yes	DCST, ICP monitoring	None		Trauma
11, f	24	aSDH		Yes	Yes	DCST, ICP monitoring	None		Non-accidental injury
12, m	26	iCH, IVH, contusion		Yes	Yes	DCST, Rickham reservoir	None	Need of VPS	Spontaneous

*N/A* not available, *d* days, *HC* hydrocephalus occlusus, *CT* computed tomography, *MRI* magnetic resonance imaging, *DCST* decompressive craniotomy in split-technique, *GCS* Glasgow Coma Scale, *f* female, *m* male, *aSDH* acute subdural hematoma, *cSDH* chronic subdural hematoma, *EDH* epidural hematoma, *tSAH* traumatic subarachnoid hemorrhage, *IVH* intraventricular hemorrhage, *VPS* ventriculo-peritoneal shunt

### Surgical technique

**Fig. 1:**
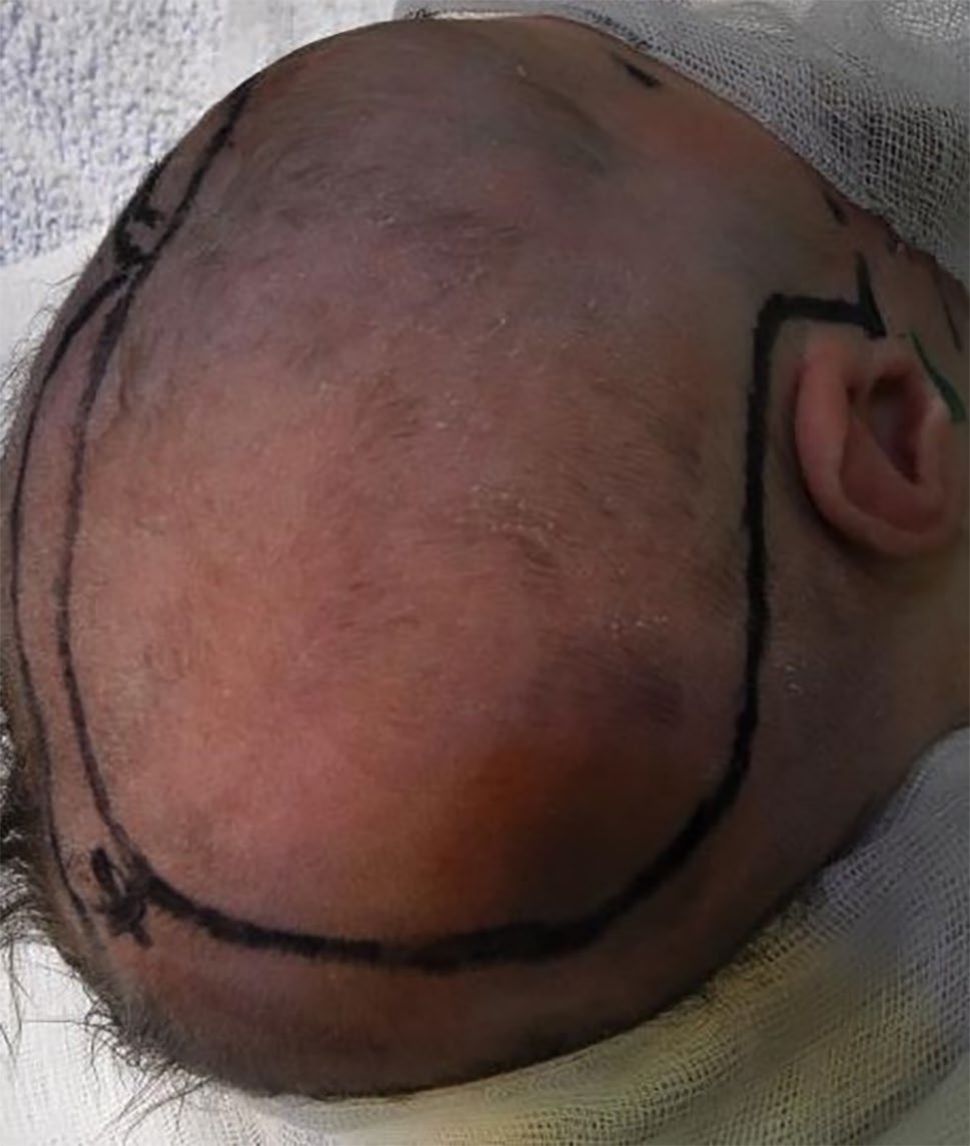
The head positioned on the vacuum mattress and rotated toward the left. The skin flap is marked in the shape of a question mark

**Fig. 2:**
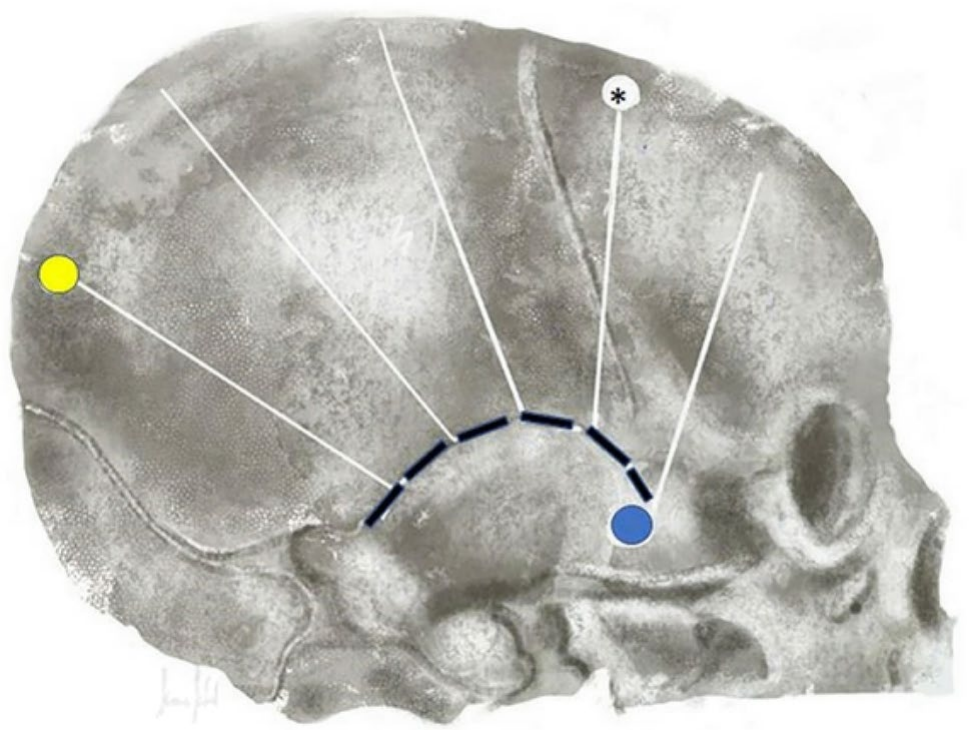
A scheme of the bone cuts after opening the skin and dissecting the temporal muscle. Three burr holes are placed on the skull. The first one is Kocher’s point (black asterisk). This burr hole is made to place an ICP probe at the end of surgery. The second is placed temporal (blue point) and the third parietal (yellow point). The dashed line shows the first temporal cut following the sutura temporo-parietalis. The white lines show the air wheel–shaped cuts of the fronto-parietal skull

**Fig. 3:**
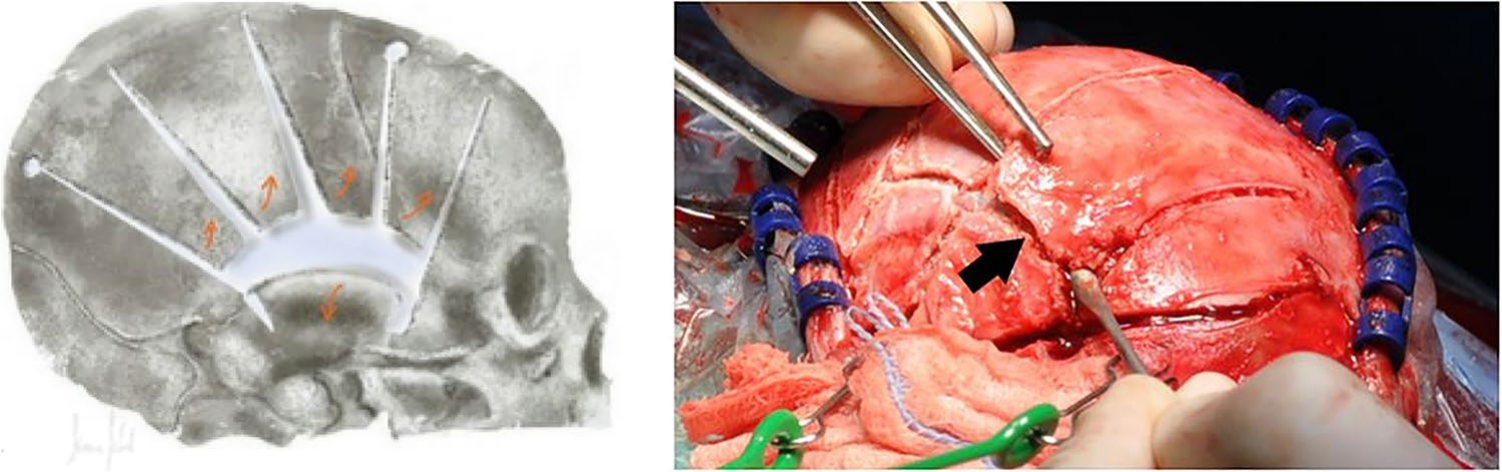
Left: This figure shows how to elevate the bone flaps (orange arrows). The light blue layer represents the dura mater. Right: The temporal bone can be dissected in the middle if it’s not flexible enough as shown in the photo (black arrow)

**Fig. 4:**
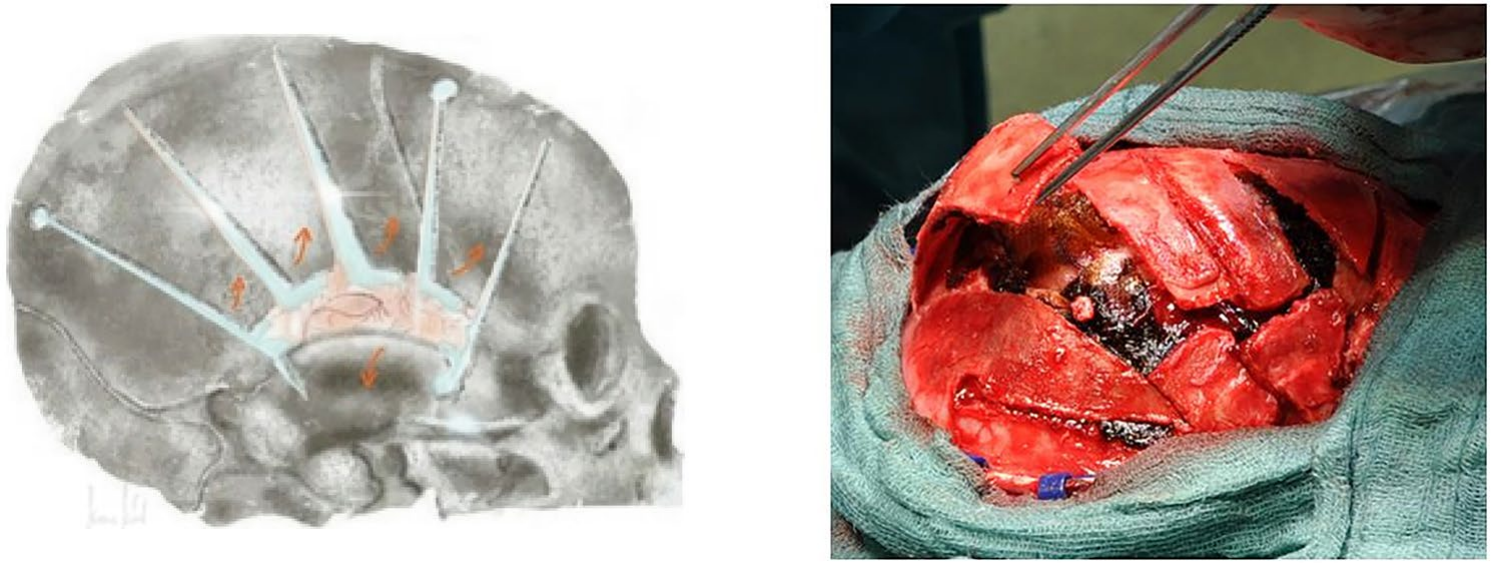
Left: This scheme shows the cut of the dura mater parallel to the cuts of the skull. Over these gaps, blood in the subdural space can be easily removed by irrigation and suction. Right: Photo shows results after decompression. We can see the flexible bone and the brain tissue with dura mater stripes covered by Tabotamp (black material on the cortex) after removing the subdural blood clot

**Fig. 5:**
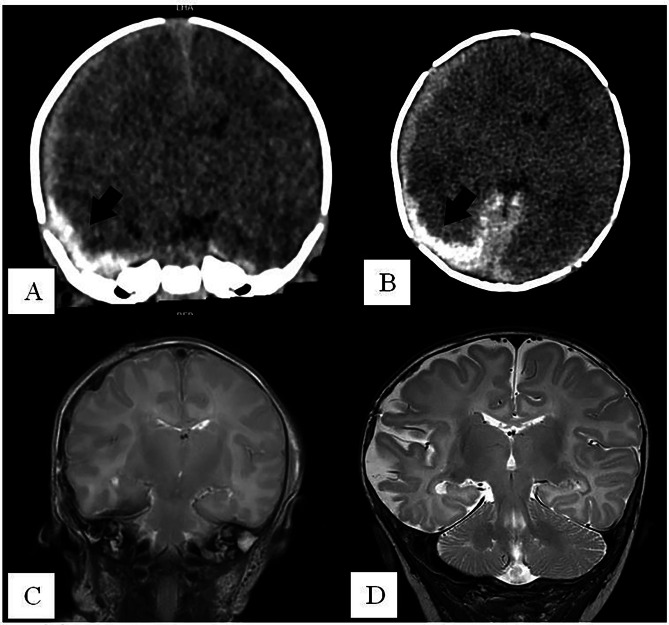
The CT scans (**A** coronal, subdural hematoma marked by black arrow; **B** axial, subdural hematoma marked by black arrow) show the subdural hematoma on the right side with midline shift to the left. **C** The postoperative MRI scan (T2, coronal) 1 day after surgery shows a decrease in midline shift. **D** The follow-up MRI scan (T2, coronal) 3 months after surgery shows the atrophy of the temporoparietal brain on the right side and the asymmetric fused bone

Surgery is performed as a standard trauma flap. The patient is placed in a supine position with the head positioned upwards with the side of the hematoma and fixed in this position with the vacuum mattress. The hair is shaved, and a trauma flap in the shape of a question mark is drawn on the skin (Fig. 1). After disinfection and covering the surgical side, the skin is opened along the marked incision. The temporal muscle is then dissected and stripped of the temporal bone to the keyhole. Three small burr holes are placed, and the basis of the fronto-temporo-parietal bone was opened (Fig. 2). The temporal bone is cut along the temporoparietal suture. The parietal bone flap is dissected in the shape of an air wheel into the direction of the sagittal sinus (Fig. 3). The underlying dura mater is also dissected in the same way (Fig. 4). In case of subdural hematomas, the blood is drained with irrigation and suction between the bone and dura mater cloths (Fig. 5). An intraoperative ultrasound, placed on the fontanelle of the infant, is performed showing no significant midline shift after decompression procedure. The bar between the open dura mater is covered by Tabotamp. After the insertion of an ICP electrode in the frontal lobe via the first burr hole (Fig. 1), the skin is closed by suturing the subcutaneous space and the skin with dissolvable stitches.

### Post-surgical course

**Fig. 6:**
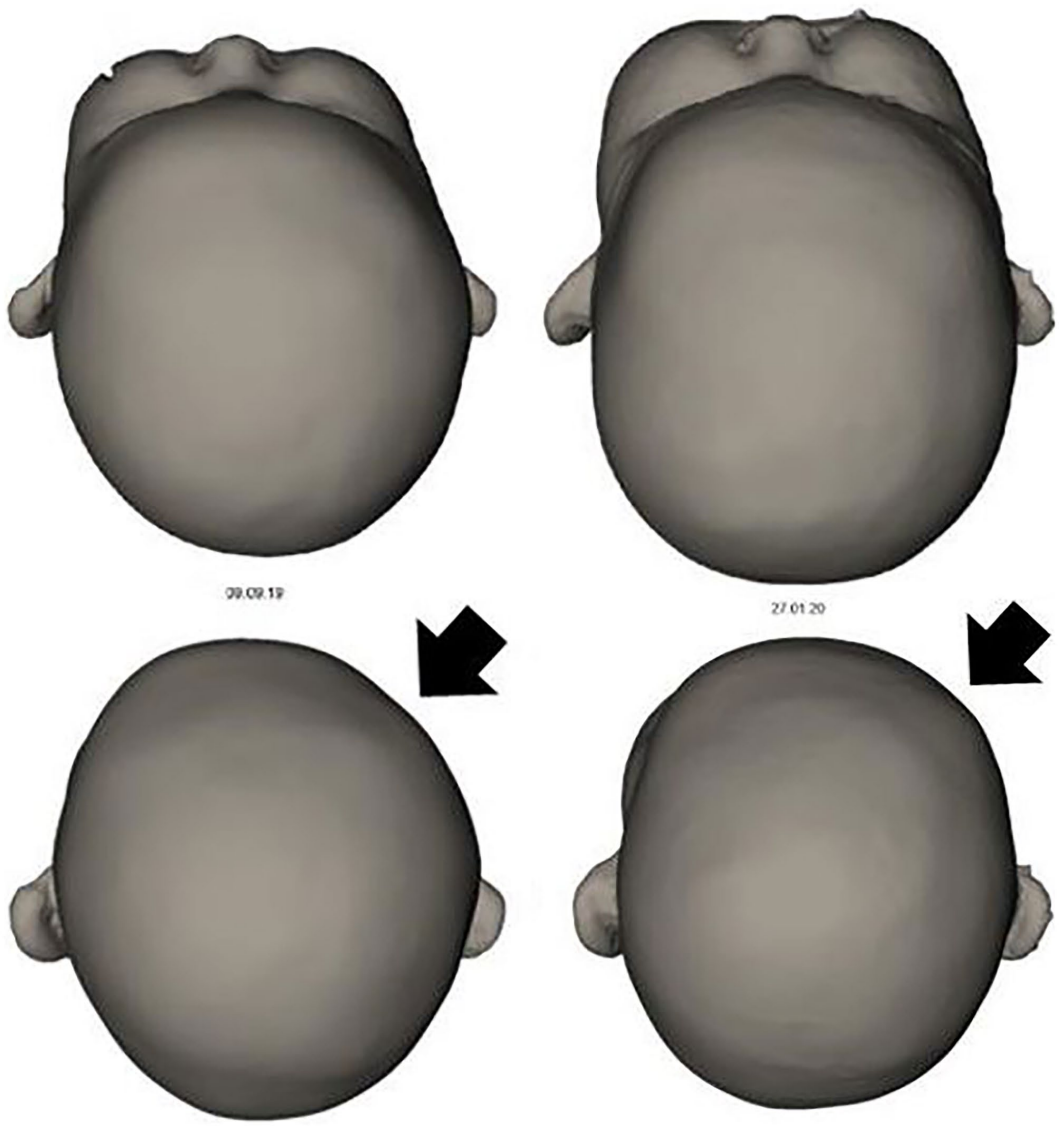
Left: The shape of the baby girl showed at the age of 4.5 months an asymmetrical fusion on the bone flaps on the surgical side (black arrow). The CVAI was 10.3%. Helmet therapy was started at the age of 5 months. Right: After a period of 4 months of helmet therapy for 23.5 h/day, the helmet could be removed. The CVAI after treatment was 3%

The patients were monitored in the NICU after the surgical procedure. The ICP decreased in all cases immediately after surgery to a normal level. The two patients without ICP monitoring were daily monitored by head ultrasound. The patients were extubated in a mean time of 29 h after DCST. All patients got a post-surgical MRI scan, which showed sufficient decompression and decreased midline shift. All treated patients did not need additional operations due to increased ICP. Two patients with intracerebral and intraventricular hemorrhage needed a shunt due to a newly developed malresorptive hydrocephalus. One was treated with a ventricular-atrial shunt by underlying peritoneal dialysis due to kidney disease on day 15 after DCST (Table 1, patient number 2). The second patient got a VPS on day 12 after DCST. Another patient developed after the surgical procedure a positional plagiocephaly with a CI of 0.64 (Fig. 6). Due to cosmetic reasons, the patient was treated with a helmet. After a period of 5.5 months of wearing the helmet, the CI increased to 0.83, and the helmet could be removed (Fig. 6).

### Surgical outcome

All patients who underwent DCST were comatose (GCS < 9) with abnormal intracerebral ultrasound and/or CT findings (significant midline shift). Two patients had pre-operative ICP monitoring showing high intracranial pressure. In all patients, the decompressive craniotomy in split-technique results in improved ICP (Table 2) within the normal range and in reduction of midline shift. To avoid child radiation exposure only MRI scans were made in the postoperative course.

**Table 2:** Summary of reported PI and ICP before and after surgery

**Patient no., sex**	**Age (d)**	**Type of hematoma**	**PI (Purcelot’s Index) (before surgery)**	**Median ICP (cmH**_**2**_**O) (before surgery)**	**Median ICP (cmH**_**2**_**O) (after surgery)**
1, m	2	aSDH	0.84	n.a.	7
2, m	339	aSDH/iCH, IVH	0.83	n.a.	n.a.
3, f	1	aSDH	0.81	n.a.	9
4, m	327	cSDH+aSDH/brain edema	n.a	19 mmHg	10
5, m	2	aSDH/iCH cerebellum	0.86	n.a.	7
6, f	163	aSDH/cSDH	n.a	18 mmHg	8
7, f	41	aSDH	0.83	n.a.	7
8, f	2	aSDH	0.84	n.a.	
9, m	332	aSDH/tSAH, brain edema	0.86	19 mmHg	8
10, m	355	EDH, aSDH	0.83	n.a.	9
11, f	24	aSDH	0.81	n.a.	7
12, m	26	iCH, IVH, contusion	0.82	n.a.	n.a.

*n.a.* not available, *d* days, *HC* hydrocephalus occlusus, *f* female, *m* male, *aSDH* acute subdural hematoma, *cSDH* chronic subdural hematoma, *EDH* epidural hematoma, *tSAH* traumatic subarachnoid hemorrhage, *IVH* intraventricular hemorrhage, *RI* Purcelot’s Index, *ICP* intra cranial pressure

In one case in our study, the shape of the skull of a baby girl showed at the age of 4.5 months an asymmetrical fusion of the bone flaps like a positional plagiocephaly. A helmet therapy was started. After a period of 4 months, the helmet could be removed (Fig. 6).

## Discussion

The central goal of the clinical management of TBI or stroke is the patient’s quality of life after receiving treatment for the injury. In cranioplasty, after decompressive craniectomy in a pediatric population, the overall complication rate is more than 40% with an infection rate of 10% and an osteolysis rate of 30%.

Grant et al. reported in their study in 2004 that the incidence of bone graft failure due to resorption correlated with a larger skull defect area. They reported that 50% of the patients with large skull defects required surgery due to osteolysis. The only significant predictor of bone resorption was time to cranioplasty. Almost one-third of the patients who got their autologous cranioplasty 6 weeks or more after craniectomy back in a surgical procedure developed osteolysis [13].

Patients with an underlying contusion were more likely to experience resorption of the autologous bone graft, as were those with comminuted skull fractures [14].

The presence of a cranial implant, a VP shunt, a gastrostomy tube, and ventilator-dependent status were statistically significant predictors of cranioplasty infection in adults [15]. Age and EVD use showed a significant association with bone resorption; similarly, the odds of resorption were higher when cranioplasty fixation was performed with suture or resorbable plates as compared to titanium plates [15].

If a resorption or an infection occurs, the treatment options are limited. In this age group, there are no allogenic bone replacements available. The common method is to perform a split craniotomy by using a bone graft from the healthy side. The consequence is a big surgery with additional trauma to the healthy side of the skull. This procedure itself can lead to complications [10–12, 14].

Park et al. described hinge and floating decompressive craniotomy in five infants with acute subdural hematoma under the age of 20 months with no operation-related complications such as infection or bone graft resorption. However, other types of intracranial hematoma or injury were not discussed in his study [16].

DCST is a sufficient method to drain aSDH and allows the brain tissue a certain amount of swelling by occupying space on the DCST side of the skull. In our study, we were able to monitor this circumstance by ICP monitoring and post-surgical MRI scans. The ICPs were, in all monitored cases, within the normal range. The MRI scans showed an improved midline shift in all cases. After a period of swelling, the brain tissue decreases in volume. The bone flaps fall behind and get rigid over time by itself. In one case in our study, the fusion of some bone flaps resulted in asymmetric fusion, which could be treated successfully with helmet therapy.

In our small study, we were able to show the sufficiency of DCST. All patients showed a decrease in intracranial pressure after surgery. Furthermore, we also recorded no additional surgery in all patients due to bone flap resorption or infection. The introduced method seems to be effective in infants with TBI. More data in a clinical setting is needed.

## Conclusions

Present data suggests that DCST has a possible beneficial role in the treatment of TBI in infants. The method is easy to implement for a trauma treatment–experienced neurosurgeon. There is no need for special instruments or new settings in the operating room. It seems to be efficient for the treatment of TBI. An additional surgery in the follow-up period compared to decompressive craniectomy could be avoided. However, the quality of evidence prevents drawing conclusions.

## Data Availability

No datasets were generated or analyzed during the current study.
